# Identification and Characterization of Zika Virus NS5 Methyltransferase Inhibitors

**DOI:** 10.3389/fcimb.2021.665379

**Published:** 2021-04-07

**Authors:** Weibao Song, Hongjuan Zhang, Yu Zhang, Ying Chen, Yuan Lin, Yanxing Han, Jiandong Jiang

**Affiliations:** State Key Laboratory of Bioactive Substances and Function of Natural Medicine, Institute of Materia Medica, Chinese Academy of Medical Sciences and Peking Union Medical College, Beijing, China

**Keywords:** Zika virus, NS5, methyltransferase, inhibitors, theaflavin

## Abstract

The recurring outbreak of Zika virus (ZIKV) worldwide makes an emergent demand for novel, safe and efficacious anti-ZIKV agents. ZIKV non-structural protein 5 (NS5) methyltransferase (MTase), which is essential for viral replication, is regarded as a potential drug target. In our study, a luminescence-based methyltransferase assay was used to establish the ZIKV NS5 MTase inhibitor screening model. Through screening a natural product library, we found theaflavin, a polyphenol derived from tea, could inhibit ZIKV NS5 MTase activity with a 50% inhibitory concentration (IC_50_) of 10.10 μM. Molecular docking and site-directed mutagenesis analyses identified D146 as the key amino acid in the interaction between ZIKV NS5 MTase and theaflavin. The SPR assay indicated that theaflavin had a stronger binding activity with ZIKV NS5 wild-type (WT)-MTase than it with D146A-MTase. Moreover, theaflavin exhibited a dose dependent inhibitory effect on ZIKV replication with a 50% effective concentration (EC_50_) of 8.19 μM. All these results indicate that theaflavin is likely to be a promising lead compound against ZIKV.

## Introduction

Zika virus (ZIKV), emerging as a global healthcare threat, belongs to mosquito-transmitted Flavivirus in the *Flaviviridae* family, which also includes Dengue (DENV), yellow fever (YFV), West Nile (WNV), Japanese encephalitis (JEV) and tick-borne encephalitis (TBEV) viruses (Miner and Diamond, 2017; Mittal et al., 2017). All the flavivirus can bring global emergencies and cause great burdens to public health. Before the French Polynesian outbreak in 2013, ZIKV was considered a mild disease (Musso et al., 2015). However, severe Guillain-Barre syndrome was found in this outbreak (Musso et al., 2014). In 2015, ZIKV was declared a global public health emergency by WHO due to its explosive spread through the Americas (Faria et al., 2016). More importantly, until now, no vaccines or drugs have been found effective for prevention or treatment of ZIKV infection (Masmejan et al., 2018). As a result, developing anti-ZIKV agents becomes an emergency.

ZIKV is a small enveloped single-stranded positive-sense RNA virus (Lindenbach and Rice, 2003). Its genome encodes a polyprotein (~3423 amino acids in length), which consists of 3 structural proteins (capsid (C), membrane (M) and envelope (E)), as well as 7 nonstructural proteins (NS1, NS2A, NS2B, NS3, NS4A, NS4B, and NS5) (Sirohi and Kuhn, 2017). Among the nonstructural proteins, NS5 is the largest enzyme including 904 amino acids (Issur et al., 2009; Piorkowski et al., 2016). It consists of 2 domains: an RNA-dependent RNA polymerase (RdRp) domain at the C-terminal and a methyltransferase (MTase) domain at the N-terminal (Elshahawi et al., 2019). The NS5 RdRp domain contributes to the viral RNA synthesis through a *de novo* initiation mechanism (Godoy et al., 2017). The NS5 MTase domain methylates the RNA cap to form N-7-methyl-guanosine and 2’-O-methyl-adenosine using S-adenosyl-L-methionine (SAM) as the methyl donor (Zhang et al., 2017).

It has shown that both N-7-methyl-guanosine and 2’-O-methyl-adenosine activities of MTase are essential for viral replication (Dong et al., 2010a; Züst et al., 2011; Züst et al., 2013). Therefore, suppression of MTase activity is a promising strategy for the development of anti-ZIKV agents. Crystal structural research revealed that the MTase core domain consisted of SAM-binding pocket, cap-binding site and positive RNA-binding site. Both SAM-binding pocket and cap-binding site of MTase have been proved essential, making the ZIKV MTase an attractive target for the development of ZIKV inhibitors (Duan et al., 2017).

The identification of target-based antiviral agents has been successful in treating viral infections, such as HIV, HCV and dengue fever (Borkow et al., 1997; Chen and Njoroge, 2010; Xu et al., 2012). Thus, the target-based drug identification strategy might be adopted in anti-ZIKV agent identification. Since ZIKV MTase is a promising drug target for anti-ZIKV agents, its inhibitors such as sinefungin and SAH (S-adenosyl-homocysteine) have been previously identified to interfere both N7 and 2′-O methylation reactions of DENV NS5-MTase (Lim et al., 2008; Chung et al., 2010; Selisko et al., 2010; Wang et al., 2018). However, due to their poor cellular permeability and non-selectivity, practical applications of these two inhibitors are limited (Lim et al., 2015). Recently, a conserved hydrophobic pocket near the SAM-binding site of ZIKV NS5 MTase was identified through structural research, paving the way of its highly specific inhibitor identifications (Dong et al., 2010b; Lim et al., 2011; Duan et al., 2017).

In this study, we first expressed and purified ZIKV NS5 MTase. Then the activity of ZIKV NS5 MTase was characterized by a luminescence-based methyltransferase assay. Meanwhile, we established a ZIKV NS5 MTase inhibitor screening system. By screening a natural product library of 228 compounds, theaflavin was identified to inhibit the activity of ZIKV NS5 MTase with a 50% inhibitory concentration (IC_50_) of 10.10 μM. Moreover, we further illustrated the inhibitory mechanism of theaflavin by molecular docking and site-directed mutagenesis. The inhibitory effect of theaflavin depended on its interaction with D146 at ZIKV NS5 MTase SAM-binding site. This direct binding was demonstrated by surface plasmon resonance assay. Anti-ZIKV activity assay showed theaflavin possessed a 50% effective concentration (EC_50_) of 8.19 μM on ZIKV replication. Theaflavin, a natural polyphenol from tea, may be a potent anti-ZIKV agent.

## Materials and Methods

### Materials

Dithiothreitol, imidazol, Tris-HCl and other common assay reagents were purchased from Sigma (St. Louis, MO, USA). The ZIKV cDNA was synthesized by Invitrogen (Waltham, MA, USA). The ZIKV RNA sequence for methyltransferase reaction was synthesized *de novo* by Genscript (Nanjing, Jiangsu, CHN). The RNA capping kit was purchased from New England Biolabs (Ipswich, MA, USA). MTase-Glo Methyltransferase Assay kit was purchased from Promega (Madison, WI, USA). HisTrap FF crude and CM5 sensor chip were purchased from GE (Uppsala, Sweden). 384-well white plates were obtained from Corning (Corning, NY, USA). Discovery Studio 2018 R2 software was provided by Accelrys (San Diego, CA, USA). The screening library was a natural product library provided by MedChem Express (Monmouth Junction, NJ, USA).

### Plasmid Construction and Protein Expression

The cDNA sequence of ZIKV NS5 MTase (nucleotide 7666 to 8463; strain ZikaSPH2015; GenBank accession number KU321639.1) was synthesized by Invitrogen (Waltham, MA, USA). The fragment was fused into the pET-30a(+) expression vector, in which N-terminal His tags were introduced both upstream and downstream. Then the reconstructed plasmid was transformed into the *E. coli* DH5α strain. Site-directed mutagenesis using the Fast Mutagenesis System (TransGen Biotech, Beijing, China) was carried out to construct plasmids carrying the ZIKV NS5 MTase mutants (D146A-MTase and K105A-MTase, respectively). BL21 was chosen for protein expression. Transformed cells were grown in LB medium containing 50 μg/mL kanamycin at 37°C till they reached A_600_ = 0.4-0.6. Protein expression was automatically induced at 20°C for 16 h in ZY medium (10 mg/mL tryptone, 5 mg/mL yeast extract, 3.55 mg/mL Na_2_HPO_4_, 3.4 mg/mL KH_2_PO_4_, 2.68 mg/mL NH_4_Cl, 0.71 mg/mL Na_2_SO_4_, 5 mg/mL glycerol, 0.5 mg/mL glucose, 2 mg/mL α-lactose monohydrate, 2 mM MgSO_4_) containing 50 μg/mL kanamycin. Cells were harvested by centrifugation. To purify, a Ni^2+^ HisTrap chelating column was used to load target protein resolved in the supernatant. Buffer A (containing 25 mM Tris, 500 mM NaCl, and 25 mM imidazole at pH 7.8) assisted to load target protein on the column. Buffer B (containing 25 mM Tris, 500 mM NaCl, and 350 mM imidazole at pH 7.8) was prepared to achieve a stepwise gradient elution. SDS-PAGE and Coomassie blue staining were used to analyze the purified protein WT-MTase, D146A-MTase and K105A-MTase. The recombinant proteins were also confirmed by Western blot using anti-His-tag antibody. Then they were stored in 20 mM Tris-HCl (pH 7.0), 500 mM NaCl, 10% glycerol, and 2 mM EDTA-2Na at -80°C until use.

### ZIKV NS5 MTase Activity Assay

NS5 WT-MTase, D146A-MTase and K105A-MTase proteins were purified as described above. The RNA sequence (5´-AGUAGUUCGCCUGUGUGAGCUGACAAACUUAGUAGUGUUUGUGAGGAUUAAUAACAAUUAACACAGUGCGAGCUGUUUCUUAGCACGAAG-3`) for methyltransferase reaction was synthesized *de novo* by Genscript (Nanjing, Jiangsu, China). Then the RNA was capped, as following: 15 μL 0.67 μg/μL RNA was heat at 65°C for 5 min, then placed at 0°C for 5 min. Subsequently, 2 μL 10× reaction buffer (500 mM Tris-HCl (pH 8.0), 50 mM KCl, 10 mM MgCl_2_ and 10 mM DTT), 1 μL 10 mM GTP, 1 μL 2 mM SAM and 1 μL 10,000 U/ml capping enzyme were added and the capping system was incubated at 37°C for 30 min (Grudzien et al., 2004). Then mixing the 167 mg/L capped RNA with 40 μM SAM solution in equal volume to produce the substrate solution. The NS5 MTase activity assay was performed in a 8 μL reaction system containing 4 μL substrate solution and 4 μL 8 mM methyltransferase solution (diluted by 4× reaction buffer: 80 mM Tris buffer, pH 8.0; 200 mM NaCl; 4 mM EDTA; 12 mM MgCl_2_; 0.4 mg/ml BSA; 4 mM DTT) using a 384-well white plate for 2 hours. Then 2 μL 0.5% TFA was added to stop the reaction. After termination of the reaction, 2 μL 6× MTase-Glo Reagent was added with an incubation of 30 min. Subsequently, 12 μL MTase-Glo Detection solution was added. After 30-minute incubation at room temperature, the released luminescence (LUM) was finally measured by luminometer (Kundaje et al., 2015).

### ZIKV NS5 MTase Inhibitor Screening

After establishing the Zika NS5 MTase activity assay, we established the high-throughput screening assay model for its inhibitors. A natural product library of 228 compounds was screened at 20 μM. The screening assay was similar to the MTase activity assay, but with some modifications. The compounds were first incubated with MTase for 1 hour at room temperature. Then the MTase activity was detected as described above. Compounds with a MTase activity inhibition ratio over 50% were picked out and were tested in a 6-dose IC_50_ mode with a two-fold serial dilution starting at 400 μM. Sinefungin, an accepted broad-spectrum MTase inhibitor, was chosen as positive control for the MTase inhibition assay. Z’ factor for the high-throughput screening model was calculated using the equation: Z’=1-3(S_p_+S_n_)/|μ_p_-μ_n_|, where μ_p_ and μ_n_ represent mean values of wells treated with 1% DMSO and wells treated with 20 μM sinefungin; S_p_ and S_n_ are the standard deviations. Data analysis was performed using GraphPad Prism 6.0 software for curve fits and IC_50_ calculation.

### Molecular Docking Between Theaflavin and NS5 MTase

The co-crystallized structure of ZIKV MTase (PDB ID: 5WXB) was downloaded from the Protein Data Bank  (Zhou et al., 2017). Water molecules and co-crystallized ligand SAH were removed from the protein. Before molecular docking, the ligand (theaflavin) and receptor (NS5 MTase) were prepared by the protocol “Prepare Ligands”, “Prepare Protein”, respectively. The docking protocol C-DOCKER was employed to predict the scoring and binding interactions between theaflavin and MTase. The docking pocket was defined based on the reported active sites of NS5 MTase, corresponding to S56, G86, W87, H110, D131, V132 and D146 (Duan et al., 2017). The docked complexes were evaluated using the C-DOCKER energy scores. The key amino acid was analyzed.

### Surface Plasmon Resonance Assay

Surface plasmon resonance assay was performed at 25°C in PBS-T running buffer (PBS containing 0.05% Tween 20 and 0.1% DMSO) using a BIAcore T200. Firstly, WT-MTase was coated onto a CM5 sensor chip. After optimizing the pH condition, the NHS/EDC amine coupling reaction was carried out for WT-MTase immobilization in 10 mM sodium acetate buffer at pH 5.0. Then different concentrations of theaflavin (ranging from 0 to 200 μM) flowed over the WT-NS5 MTase immobilized chip. Response unit (RU) was used to reflect the binding activity between theaflavin and WT-MTase. Experiment data were analyzed by the BIAcore evaluation software package and the compound-protein interaction was illustrated by the equilibrium dissociation constant (*K*_D_). The same method was employed to examine the interaction between D146A-MTase and theaflavin.

### *In Vitro* Anti-ZIKV Activity

The anti-ZIKV activity of theaflavin was evaluated by a cell viability assay based on ZIKV-induced cytopathic effect (CPE) using chloroquine as a positive control (Han et al., 2018; Lin et al., 2019). Monolayers of Huh-7 cells were cultured in six well tissue culture plates and incubated under 5% CO_2_ at 37°C. Before infected by the ZIKV (SMGC-1) (obtained from the Institute of Microbiology, Chinese Academy of Sciences, Beijing, China), Huh-7 cells were pre-treated with chloroquine or theaflavin at serial concentrations (0-128 μM) for 1 hour. After infected with ZIKV at a multiplicity of infection (MOI) of 0.1, the inoculum was removed and was replaced with medium containing chloroquine or theaflavin. The plates were incubated for 72 h. Then the cell viability was measured by a CellTiter-Glo Luminescent cell viability kit (Promega, WI, USA) according to the manufacturer’s instructions at 72 h post-infection (hpi). Wells containing uninfected cells with no drugs served as the control (100%). Antiviral activity was determined based on the percentage of cell viability of cultures treated with drugs compared with the control. The concentration of a compound achieving 50% protection against virus induced cell death was defined as the 50% effective concentration (EC_50_). EC_50_ value was calculated by nonlinear regression using GraphPad Prism 6.0 software.

### Quantitative Reverse Transcription Polymerase Chain Reaction (qRT-PCR) Assay

ZIKV RNA was quantitatively determined by qRT-PCR. Huh-7 cells were treated with chloroquine or theaflavin at serial concentrations (0-128 μM) for 1 hour before infection by ZIKV. After ZIKV infection, Huh-7 cells were incubated with medium containing chloroquine or theaflavin for 72 h. The cell supernatants were collected at 72 hpi for viral RNA copies measurement using qRT-PCR. Total RNA was extracted using Trizol reagent (Invitrogen, MA, USA). Then qRT-PCR was performed using primers as follows: ZIKV-forward: 5′-TGCCCAACACAAGGTGAAGC-3′, ZIKV-reverse: 5′-CTCTGTCCACTAAYGTTCTTTTGC-3′. Amount of viral RNA was quantitatively assayed using RNA UltraSense One-step quantitative RT-PCR kit (Invitrogen, MA, USA) according to the manufacturer’s instructions. The significance level between two groups was calculated using t-test. P value < 0.5 was considered to show a significant difference.

### CCK-8 Assay

Cytotoxicity of theaflavin was determined using CCK-8 assay. Huh-7 cells were cultured under 5% CO_2_ at 37°C before injected into the 96-well plates. Then two-fold serially diluted theaflavin from 1 to 128 μM was added in three replicates. After 24-hour incubation, medium was discarded and CCK-8 reagent was added. Detection was carried out after 2.5 hours incubation at 37°C.

## Results

### Purification and Characterization of ZIKV NS5 MTase

**Figure 1:**
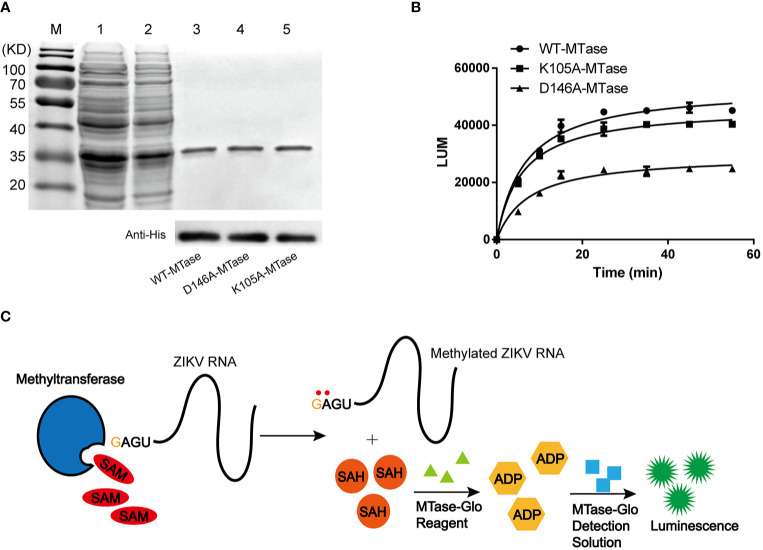
Purification and characterization of ZIKV NS5 MTase. **(A)** Purification of NS5 MTase and its mutants. SDS-PAGE (top) and Western blot (bottom) using anti-His-tag antibody analysis of the recombinant proteins. Lane M, protein marker. Lane 1, total cellular proteins containing induced recombinant WT-MTase. Lane 2, supernatant of cellular lysate containing induced recombinant WT-MTase. Lane 3, purified recombinant WT-MTase. Lane 4, purified recombinant D146A-MTase. Lane 5, purified recombinant K105A-MTase. **(B)** Activity of recombinant WT-MTase, D146A-MTase and K105A-MTase. Luminescence-based methyltransferase assay was used. The time course experiment was repeated three times. **(C)** Schematic for the principle of the luminescence-based methyltransferase assay.

The NS5 MTase recombinant protein was expressed in a soluble form and was purified using Ni^2+^ HisTrap chelating columns. In SDS-PAGE analysis, the purified MTase showed a molecular weight of approximately 35 kDa (**Figure 1A**). The recombinant protein was also confirmed by Western blot using anti-His-tag antibody (**Figure 1A**).

The NS5 MTase activity assay was carried out by mixing NS5 MTase with capped virus RNA and SAM in the assay buffer. During the reaction, methyls of SAM were transferred onto the GTP-cap and adjacent adenine, leaving SAH as a by-product of the reaction. The principle of MTase activity assay is the indirect detection of SAH. First, addition of MTase-Glo Reagent converted the generated SAH to ADP. Subsequently, addition of MTase-Glo Detection Solution converted ADP to ATP, which is detected via a luminescence emission reaction. The released luminescence signal was used to quantify the enzyme activity. In general, the increase of SAH corelated with the enhancement of the LUM signal. The mechanism of the MTase activity assay was shown in **Figure 1C**. Time course experiment showed the enzymatic activity of WT-MTase increased (~50, 000 LUM) as the reaction time extended (0-60 min) (**Figure 1B**). It approached the stable phase after an incubation of 35 min.

### Inhibitory Effects of Theaflavin on ZIKV NS5 MTase Activity

**Figure 2:**
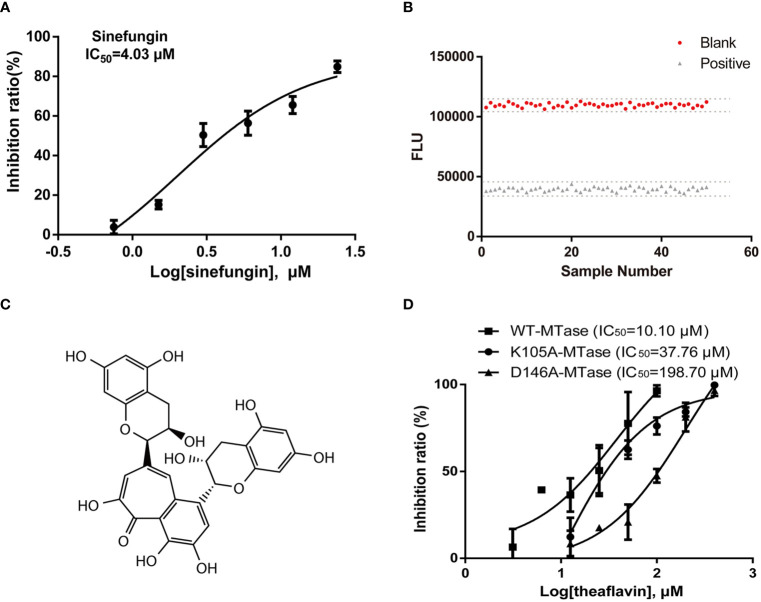
Screening assays for ZIKV NS5 MTase inhibitors and identification of the hit compound theaflavin. **(A)** Inhibition of MTase by the positive control sinefungin. Two-fold dilutions (0.75-24 μM) of sinefungin were used. The MTase activity of the DMSO control was set as 100%. **(B)** Z’ factor of the luminescence-based assay. MTase was incubated with 20 μM positive control sinefungin or 1% DMSO for 1 h at room temperature. Then the luminescence intensity was measured and Z’ factor was calculated as described. **(C)** Structure of theaflavin. **(D)** WT-MTase and mutant MTases inhibition assays. Half maximal inhibition concentration (IC_50_) values were calculated from dose-response curves in three independent experiments using GraphPad 6.0.

Based on the methyltransferase assay, we established the high-throughput screening assay. Sinefungin, a broad-spectrum MTase inhibitor, was used as a reference compound. As is shown in **Figure 2A**, sinefungin exhibited a robust dose-dependent inhibition to NS5 MTase (IC_50_ = 4.03 μM) in the screening assay. Then, we determined the Z’ factor of the luminescence-based screening for ZIKV NS5 MTase inhibitors in the 384-well plate using 20 μM sinefungin or 1% DMSO. The Z’ factor of the assay is 0.86 (**Figure 2B**).

The initial screening was carried out using methyltransferase assay to screen a natural product library of 228 compounds at 20 μM. Two compounds, theaflavin and chitosan, with MTase inhibitory activities over 50% were selected. Then, theaflavin and chitosan were tested for IC_50_ by methyltransferase inhibitory assay. We found theaflavin had an IC_50_ of 10.10 μM (**Figure 2D**), better than chitosan with an IC_50_ of 26.99 μM. Therefore, theaflavin was selected for the further study. The structure of theaflavin was shown in **Figure 2C**.

### Molecular Docking Between Theaflavin and ZIKV NS5 MTase

**Figure 3:**
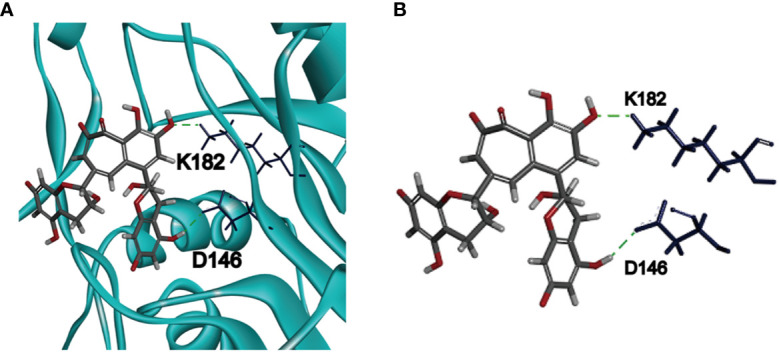
Molecular docking of ZIKV NS5 MTase and theaflavin. **(A)** The predicted pose of theaflavin binding to the active pocket of ZIKV NS5 MTase. Theaflavin is represented by a stick model (grey, carbon atoms; deep red, oxygen atoms; light grey, hydrogen atoms). The deep blue sticks represent the amino acids interacting with theaflavin. **(B)** The detailed intermolecular bonds between ZIKV NS5 MTase and theaflavin. The hydrogen bond between D146 and the hydrogen atom of theaflavin is green. Hydrogen bond is also observed between K182 and oxygen atom of theaflavin.

To investigate the binding mechanism between theaflavin and MTase, we performed molecular docking using CDOCKER protocol of Discovery Studio 2018R2 software. The crystal structure of MTase was acquired from Protein Data Bank (PDB: 5WXB) with a resolution at 1.76 Å. The binding mode getting the highest score of CDOCKER energy was shown in **Figure 3A**. The detailed binding information showed that theaflavin bound to MTase by two conventional hydrogen bonds (H-bonds) (**Figure 3B**). One is formed by the hydroxyl group of theaflavin with D146, a key amino acid for ZIKV NS5 MTase activity (Duan et al., 2017). The other is formed by the oxygen atom of the theaflavin with K182 residue, which located out of the active center. Therefore, we inferred that the inhibitory effect of theaflavin to MTase activity is dominated by the H-bond interaction between D146 and theaflavin.

### Construction of ZIKV NS5 MTase Mutations and Evaluation of Theaflavin Activity

Based on the molecular docking results, we conducted site-directed mutagenesis to further investigate the inhibition mechanism of theaflavin to MTase. We mutated D146 to A146 and carried out a bioactivity analysis. Meanwhile, we mutated K105 (the amino acid located outside of the active center) to A105, which was used as a negative control. The mutant proteins were named D146A-MTase and K105A-MTase, respectively. Both D146A-MTase and K105A-MTase proteins were purified (**Figure 1A**). The methyltransferase activity of K105A-MTase decreased slightly compared with WT-MTase, but D146A-MTase activity decreased sharply compared with WT-MTase (**Figure 1B**). To determine the inhibition mechanism of theaflavin, both D146A-MTase and K105A-MTase were used to evaluate the inhibition activity of theaflavin. In the luminescence-based methyltransferase assay, the IC_50_ values of theaflavin for D146A-MTase and K105A-MTase were 198.70 μM and 37.76 μM, respectively (**Figure 2D**). We observed that theaflavin had a 20-fold IC_50_ value increase when the receptor was changed from WT-MTase to D146A-MTase. However, IC_50_ value raised slightly, when WT-MTase was changed to K105A-MTase. Combining the results of molecular docking and protein mutant assays, we speculated that theaflavin bound to D146 to inhibit NS5 MTase activity.

### Confirming the Interaction Between Theaflavin and ZIKV NS5 MTase

**Figure 4:**
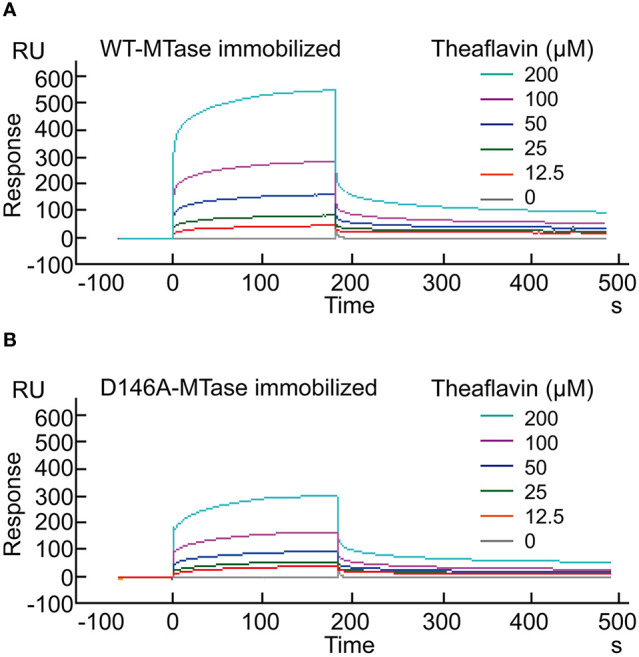
Surface plasmon resonance analysis for the binding of theaflavin to WT-MTase and its mutant. **(A)** Binding of theaflavin to WT-MTase protein. Various concentrations (0-200 μM) of theaflavin were injected into the chamber of a WT-MTase coated sensor chip. The change of response units (RU) is shown. **(B)** Binding of theaflavin to D146A-MTase protein.

The SPR technique is considered one of the most accurate analytical tools for detection of biomolecular interactions between proteins and small molecules. Thus, the interaction between theaflavin and ZIKV NS5 MTase could be measured by SPR. Purified WT-MTase or D146A-MTase was coated onto the BIAcore CM5 sensor chip, respectively. Then they were exposed to various concentrations of theaflavin to measure the binding activity by RU. Evident increase in RU inferred theaflavin could bind with WT-MTase in a dose-dependent (0-200 μM) manner (**Figure 4A**). When D146A-MTase was chosen as the analyte, there was an obvious decrease in RU compared with WT-MTase (**Figure 4B**).

In SPR analysis, we use *K*_D_ (equilibrium dissociation constant) to measure the interaction intensity between the compound and the protein. Data showed theaflavin could bind with WT-MTase with the *K*_D_ equaled to 6.44×10^-5^ μM. However, the *K*_D_ increased 4 times when the protein coated on CM5 censor chip was changed from WT-MTase to D146A-MTase (25.19×10^-5^ μM), which indicated the interaction intensity between theaflavin and D146A-MTase decreased comparing with that between theaflavin and WT-MTase. Combining the results of MTase activity assay, molecular docking and SPR analysis, we speculated theaflavin inhibited WT-MTase activity by binding to the key amino acid D146.

### Anti-ZIKV Activity of Theaflavin

**Figure 5:**
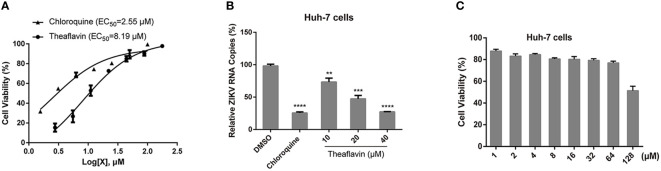
The theaflavin inhibits ZIKV infection with low cytotoxicity. **(A)** Dose dependent inhibition of ZIKV by theaflavin in Huh7 cells. Chloroquine was used as a positive control. **(B)** qRT-PCR analyses of viral RNA inhibition by theaflavin in Huh7 cells. ^**^P < 0.01, ^***^P < 0.001, ^****^P < 0.0001. Data were shown as the mearn ± SD (N = 3). **(C)** Cytotoxicity of theaflavin. Huh7 cells were treated with different concentrations of theaflavin (1-128 μM). The cell viability was measured by CCK-8 assay.

The anti-ZIKV activity of theaflavin was first assessed by cell viability assay based on ZIKV-induced cytopathic effect (CPE). It’s a simple and fast method to evaluate the anti-ZIKV agents by measuring its ability to inhibit ZIKV-induced CPE. Now it has been used in drug discovery to preliminarily evaluated the anti-ZIKV activities (Shan et al., 2016; Xie et al., 2016; Han et al., 2018). After 72 hpi, cell viability was measured using a CellTiter-Glo Luminescent cell viability kit, a luminescent-based assay that quantifies the amount of ATP in metabolically active cells. Chloroquine was used as a positive control. It was shown that chloroquine and theaflavin could restore Huh7 cell viability or protect from ZIKV induced CPE in a dose dependent manner. The EC_50_ of chloroquine and theaflavin was 2.55 μM and 8.19 μM respectively, suggesting a potent anti-ZIKV activity of theaflavin (**Figure 5A**). To further monitor viral RNA production in Huh7 cells after ZIKV infection, we performed the virus yield reduction assay of theaflavin by qRT-PCR. As expected, chloroquine and theaflavin significantly reduced viral RNA copy numbers in a dose dependent manner. Treatment with 20 μM theaflavin led to more than 50% reduction of viral RNA detected in the supernatant (**Figure 5B**). Combined the cell viability assay with the virus yield reduction assay by qRT-PCR, we preliminarily determined the anti-ZIKV activity of theaflavin. CCK-8 assay indicated that CC_50_ (50% cytotoxic concentration) of theaflavin to Huh7 cells was higher than 128 μM, which is 10 times higher than its EC_50_ to ZIKV infected Huh-7 cells, indicating the inhibitory effect of theaflavin to Huh7 was not due to its cytotoxicity (**Figure 5C**).

In all, these results revealed that theaflavin could target D146 of ZIKV NS5 MTase to inhibit ZIKV infection.

## Discussion

Currently, several promising ZIKV drug targets encoded by ZIKV or present in host cells have been identified. Compounds that could prevent the progression of ZIKV infection or interfere with the function of ZIKV proteins were considered the potential anti-ZIKV agents. Among these, inhibitors targeting ZIKV proteins were significant. NS2B/NS3 protease inhibitor temoporfin (Li Z. et al., 2017), NS3 helicase inhibitor suramin (Albulescu et al., 2017), envelope glycoprotein inhibitor nanchangmycin (Rausch et al., 2017), NS5 RdRp inhibitor sofosbuvir (Xu et al., 2017) and NS5 methyltransferase inhibitor sinefungin (Coutard et al., 2017) were all promising anti-ZIKV drugs. Although considerable resources have been invested into anti-ZIKV drug discovery, no drugs are available in clinic. The main reason for this is their high inhibition concentration to ZIKV or their cytotoxicity (Xie et al., 2017). Among these existing drug targets, ZIKV NS5 MTase was one of the most promising ones.

The crystal structure of ZIKV NS5 MTase revealed that the SAM-dependent domain which contained a binding site for SAM could be a drug target (Duan et al., 2017). Currently, several inhibitors targeting NS5 MTase SAM-dependent domain have been identified. Sinefungin, an analogue of SAM, has been shown to inhibit NS5 MTase of DENV and WNV with the IC_50_ of approximately 0.63 μM and 14 μM, respectively (Dong et al., 2008). In this research, we found sinefungin could inhibit ZIKV NS5 MTase with an IC_50_ of 4.03 μM. Therefore, we selected sinefungin as a positive control for ZIKV NS5 MTase inhibitor screening. However, sinefungin exhibited anti-virus activities with the EC_50_ of 77 μM for DENV, 27 μM for WNV and > 50 μM for ZIKV (Dong et al., 2008; Tao et al., 2018). The weak anti-ZIKV activity made it insufficient to be a candidate. Interestingly, theaflavin showed better activity against ZIKV with an EC_50_ of 8.19 μM, leading it a more potent anti-ZIKV hit. In addition to the SAM analogues, another SAM-competitive inhibitor NSC 12155, obtained from virtual screening in a compound library, showed inhibitory activities toward NS5 MTase of WNV and YFV *in vitro* with the IC_50_ ranging from 0.50 to 3.00 μM. Antiviral efficacy of NSC 12155 toward WNV, DENV-2 and JEV was also observed in cell-based assays with the EC_50_ ranging from 1.00 to 7.00 μM (Brecher et al., 2015). However, NSC 12155 showed no inhibitory activity toward ZIKV. Our data showed that theaflavin inhibited ZIKV NS5 MTase activity with an IC_50_ of 10.10 μM, as well as the EC_50_ of 8.19 μM in the *in vitro* anti-ZIKV activity assay. Theaflavin was able to interfere with NS5 MTase activity by directly binding to the key amino acid D146, which located in the SAM-binding pocket. Compared with compound NSC 12155, theaflavin exhibited a similar but more distinct inhibition mechanism. Moreover, NSC 12155 was screened from a cancer therapeutic compound library and exhibited higher cytotoxicity compared with theaflavin. In conclusion, it is obvious that theaflavin shows advantages over NSC 12155.

For anti-ZIKV drug development, repurposing clinically approved drugs is an effective approach. Chloroquine, a widely used antimalarial drug, is found to significantly inhibit ZIKV infection *in vitro*, with an EC_50_ of 1-5 μM (Li C. et al., 2017; Han et al., 2019). Chloroquine inhibits the changes of low pH-induced conformation required for the fusion of the ZIKV envelope protein with the endosomal membrane (Delvecchio et al., 2016). It blocks ZIKV replication at an early stage (Persaud et al., 2018). It is known that RNA viruses mutate frequently, which leads to a need for novel antiviral agents with distinct mechanism. Due to the essential role of ZIKV NS5 MTase, development of its inhibitor is an attractive strategy. In this research, we identified theaflavin from the natural product library. It is the first natural compound reported to bind with the active residue (D146) to inactive the ZIKV NS5 MTase. The low cytotoxicity of natural compound might make theaflavin a promising compound in anti-ZIKV drug development.

As one of the components from tea, theaflavin is a polyphenol with various biological properties, such as anti-viral, anti-bacterial, anti-metabolic syndrome and anti-tumor activities (Sahoo et al., 2016; Takemoto and Takemoto, 2018). By interfering with the p53 function, theaflavin regulated multiple hallmarks of carcinogenesis, which corelated with its anti-tumor activity (Mohanty et al., 2012). It was reported that theaflavin could increase the expression of some metabolism-related genes, significantly enhancing the systemic energy expenditure that contributed to its anti-metabolic syndrome activity (Kudo et al., 2015). For anti-bacterial activity, theaflavin exhibited antimicrobial effects by interfering with the biofilm conformation (Kong et al., 2015). Recently, scientists paid more attention to its anti-viral activities. Sahoo et al. found it was a natural inhibitor against influenza A (H1N1) neuraminidase (Sahoo et al., 2016). It also showed anti-herpes simplex virus activity with an EC_90_ of 50 μM (de Oliveira et al., 2015). Most importantly, theaflavin exhibited significant inhibitory activity toward SARS-CoV-2 in a dose-dependent manner by targeting the 3CL-protease, which was essential for the cleavage of its polyprotein to make individual proteins functional and absolutely needed for replication of the virus (Jang et al., 2020). Moreover, *in silico* docking showed theaflavin could form hydrogen bonds with SARS-CoV-2 RdRp at residues Asp452, Arg553 and Arg624, indicating its potential anti-SARS-CoV-2 activity as well (Lung et al., 2020). However, there have been no reports of theaflavin’s anti-ZIKV activity until now. Like we mentioned above, we conducted that theaflavin inhibited ZIKV NS5 MTase by binding to the active amino acid D146 in SAM-binding site. Theaflavin is the first natural compound reported to directly bind to the active site D146 of ZIKV NS5 MTase to inhibit ZIKV replication.

Hopefully, theaflavin can be developed as an effective anti-ZIKV agent by structure-based optimization and rational design in the future. This research may help us to find new anti-ZIKV drug candidates and provide further insights into anti-ZIKV drug development. Moreover, the results in this research have also provided some deep insights into COVID-19 drugs discovery, as MTase is also a highly viable drug target for SARS-CoV-2.

## Data Availability Statement

The original contributions presented in the study are included in the article/supplementary material. Further inquiries can be directed to the corresponding authors.

## Author Contributions

JJ, YH and YL planned and designed the study. WS, HZ, YZ and YC performed the experiment and analyzed the data. YL, WS and JJ wrote and revised the manuscript. All authors contributed to the article and approved the submitted version.

## Funding

This work was supported by National Natural Science Foundation of China (No. 81773784), CAMS Major Collaborative Innovation Project (No. 2016-I2M-1-011), Beijing Nova Program (No. Z181100006218075), Basic Scientific Research Program of CAMS (No. 2018RC350005), and Drug Innovation Major Project (No. 2018ZX09711001-002-002).

## Conflict of Interest

The authors declare that the research was conducted in the absence of any commercial or financial relationships that could be construed as a potential conflict of interest.
